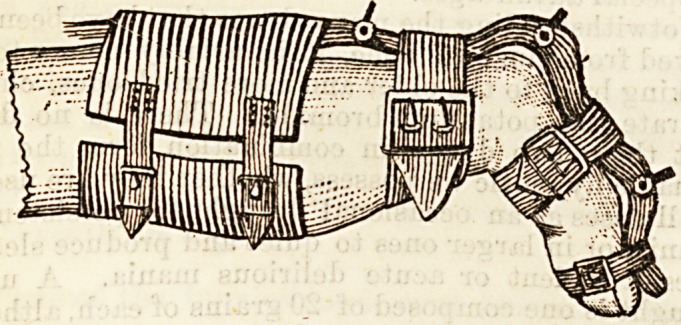# Treatment of Contraction of the Fingers

**Published:** 1893-12-02

**Authors:** 


### ROYAL ORTHOPÆDIC HOSPITAL.

### Treatment of Contraction of the Fingers.

*Contraction of the Fingers.*—This takes place from a variety of causes. Among them may be mentioned the contraction of scars left by burns, or other local injuries, the contraction of the palmar fascia, the typical example of this being the condition known as Dupuytren's Contraction. Other common causes of contraction of the fingers are affections of the tendons, or deep ligamentous structures, and stiffness or disease of the joints.

The treatment of these cases naturally varies according to the cause. If the contraction be due to adhesions in or about the joints, attempts are made to break them, by manipulation, if necessary under gas and the range of movement thus obtained is increased by means of active and passive movement. In some cases it is found that little good can be done in this way, as the adhesions are too strong to break. The attempt is then made to straighten the finger by means of the screw apparatus shown in the diagram. This consists of a splint fixed along the back of the hand and the affected finger or fingers. Opposite the knuckles are rack and pinion joints in the splint, by which it can be bent into the desired shape. The apparatus is applied to the finger in the deformed position, and then by means of the rack it is forced into as good a position as is possible without too great pressure. The great trouble that always occurs with this instrument is that sores are very liable to form at the back of the fingers. It must, therefore, be removed twice a day, and if it is found necessary the pressure be relaxed for a short time. When the splint is off, the joint is always well worked before it is replaced. After a few weeks there is generally a considerable improvement in the condition of the finger, and the apparatus is then replaced by a lighter form of splint. This usually consists of a soft iron splint, which is padded and then bent to the required shape.

When the contraction is due to the scar left from a burn, it is generally treated by means of massage and frequent working and stretching. In this way it is found that in all but the very severe cases a satisfactory amount of movement can be obtained. If not, the scar is divided or some sort of plastic operation is performed.

*Dupuytren's Contraction.*—This curious deformity is frequently seen at the Royal Orthopædic Hospital. It is caused by a thickening and contraction of the palmar fascia, commencing in the palm of the hand where the digital prolongations are given off. Its origin is uncertain. It is regarded by some of the surgeons as due to irritation, as, for instance, when it occurs among shopmen who constantly break twine by jerking it while it is wrapped around the finger. Others, again, regard it simply as a manifestation of rheumatism or gout. It is treated by subcutaneous division of the contracted band. A narrow tenotomy knife is inserted between the skin and the fascia, and then the band is divided downwards. It is sometimes necessary to divide the band in several places, the points chosen being a little above the transverse crease of the hand; between the transverse crease and the web of the fingers, and on either side dividing the lateral prolongations as they pass down to become connected to the periosteum of the first phalanx. These last two points are, perhaps, the most important of all. It is not necessary to divide the flexor tendon, as this remains securely fixed in its sheath and far removed from the tight fascial band. After division of the band the finger is extended by means of the screw instrument described above, and afterwards it is treated precisely the same.

*Congenital Contraction of the Fingers.*—This deformity although called congenital is usually little marked at birth, but gradually increases in severity during the first fifteen or sixteen years of life. Like Dupuytren's contraction, it is an affection of the palmar fascia, the chief difference being that it is very liable as time goes on to affect several fingers, and that in any case it specially interferes with the fibres prolonged from the fascia to the skin of the fingers. It is treated like Dupuytren's already described, special care being given to divide the fibres passing to the skin.

*Webbed Fingers.*—Of this curious malformation a considerable number of cases are seen at the Royal Orthopædic Hospital. It may affect all the fingers, but is perhaps most commonly met with in uniting the first and second, or the second and third. The union is usually formed of a thick band of tissue extending up to the tips of the fingers, but it may stop short of this. These cases are generally treated by means of a silver ring, or of the clamp, which is a little more convenient. A hole is made through the connecting band where the flatural cleft between the fingers should begin, and through the hole is pushed the wire of the ring or the rod of the clamp. The case is left until the raw surface has healed, leaving the hole patent. The band is then split up, and the fingers are carefully kept apart until the wound has scarred over.

The explanation of the treatment adopted is, that if the connecting tissue be simply divided it is impossible to keep the fingers thus separated from uniting from the angle outwards, and thus reproducing the webbed condition, while, if by means of the wire the point actually forming the angle has been already healed, half the trouble is avoided, and a good result can generally be obtained.

## Figures and Tables

**Figure f1:**